# The SG90 cohort of the oldest-old in Singapore

**DOI:** 10.1007/s10654-025-01275-0

**Published:** 2025-07-19

**Authors:** Lihuan Guan, Lei Feng, Anderson Li Yang Khoo, Kaisy Xinhong Ye, Roger Ho, Tze Pin Ng, Anis Larbi, Brian K. Kennedy, Woon-Puay Koh, Yap Seng Chong, Andrea B. Maier

**Affiliations:** 1 Healthy Longevity Translational Research Programme, Yong Loo Lin School of Medicine, National University of Singapore, Singapore, Singapore; 2 NUS Academy for Healthy Longevity, National University Health System, 10 Medical Drive, Singapore, 117597 Singapore; 3 Centre for Healthy Longevity, National University Health System, Singapore, Singapore; 4 Department of Psychological Medicine, Yong Loo Lin School of Medicine, National University of Singapore, Singapore, Singapore; 5 Institute for Health Innovation and Technology (iHealthtech), National University of Singapore, Singapore, Singapore; 6 Yong Loo Lin School of Medicine, National University of Singapore, Singapore, Singapore; 7 Singapore Immunology Network (SIgN), A*STAR, Singapore, Singapore; 8 Department of Medicine, Faculty of Medicine and Health Sciences, University of Sherbrooke, Sherbrooke, QC Canada; 9 Department of Biochemistry, Yong Loo Lin School of Medicine, National University of Singapore, Singapore, Singapore; 10 A*STAR Institute for Human Development and Potential, Singapore, Singapore; 11 Department of Obstetrics & Gynaecology, National University Health System, Singapore, Singapore; 12 Human Potential Translational Research Programme, Yong Loo Lin School of Medicine, National University of Singapore, Singapore, Singapore; 13 Department of Human Movement Sciences, Amsterdam Movement Sciences, Faculty of Behavioural and Movement Sciences, Vrije Universiteit Amsterdam, Amsterdam, Netherlands

**Keywords:** Ageing, Biomarkers, Oldest-old, Cohort

## Abstract

The global population is ageing rapidly. While genetics, lifestyle, and environment are known contributors to healthspan, most insights are drawn from Western cohorts, leaving Asian populations underrepresented despite unique biological, lifestyle, and cultural factors. The SG90 cohort study aimed to fill knowledge gaps in healthy ageing by identifying modifiable medical, biological, lifestyle, psychological, behavioural, and social factors that contribute to longevity in the oldest-old. The study recruited 1,158 participants aged 85 and above from the Singapore Chinese Health Study (SCHS) and Singapore Longitudinal Aging Study (SLAS) between 2015 and 2021. Data collection involved face-to-face interviews to obtain sociodemographic, lifestyle, sleep, functional status, quality of life, medical conditions and healthcare economics information, along with clinical assessments covering physical examinations, anthropometry, physical performance, cognition, and mental health. Biospecimens, including blood, saliva, stool, urine, toenails, hair, and skin tape strips were collected to support extensive multi-omic and cellular analyses. Participants, primarily female (64.5%) and Chinese (97.5%) with a median age of 87 years [interquartile range (IQR): 86–89], were mostly non-smokers (72.1%) and infrequent alcohol consumers (94.9%), with 66.5% exercising regularly. Functional assessments indicate high independence, with median Basic activities of daily living (BADL) and Instrumental ADL (IADL) scores of 20 (IQR: 19–20) and 14 (IQR: 11–16), respectively. 36% of participants rated their self-reported health as good to excellent. The SG90 cohort study offers a comprehensive clinical and biological data resource on healthy ageing among Asia’s oldest-old, laying a foundation for targeted interventions to promote healthy longevity and quality of life.

## Introduction

The world population is ageing fast and the number of oldest-old has been increasing in the past decades. In 2020, more than 147 million people globally were between the ages of 80–99 years, accounting for 1.9% of the global population, and one in a thousand persons live to 100 years old or older [1]. A nation or region is considered “ageing,” “aged,” or “super-aged” when the percentage of individuals aged 65 and above exceeds 7%, 14%, and 20%, respectively. Singapore is one of the countries with the fastest population ageing [2]. Singapore surpassed the 14% mark in 2021, officially categorizing it as an “aged” nation, and is expected to reach the “super-aged” category by 2030 with one in four Singaporeans aged 65 years and over [3]. The percentage of oldest old individuals who are aged 85 years and older among the population of 65 years older in Singapore is projected to increase from 9.2% in 2020 to 26.8% in 2060 [4].

While our life expectancy is increasing, our likelihood of living without chronic diseases or disabilities of ageing, termed healthspan, has not increased to the same extent [5]. The gap between lifespan and healthspan is increasing and estimated to be around 9 years [6]. A greater number of years with diseases and disabilities is not only associated with a higher healthcare cost [7], but also a lower quality of life [8]. This is especially relevant to Singapore as the current gap in Singapore averages is around 11 years, which is greater than the global average [9]. Ageing processes are predominantly studied in animal models and findings cannot be directly translated to humans without clinical studies [10]. Therewith, epidemiological studies representing a myriad of factors involved in healthy longevity are needed. Cohort studies enable the collection of comprehensive data, offering preliminary insights into potential intervention targets that can subsequently be tested in interventional studies to establish causal relationships [11]. There are several oldest-old cohort studies including Western populations, such as the Leiden 85-plus cohort [12], the Newcastle 85 + study [13], and the 90 + study from US [14]. In Eastern populations, the Chinese Longitudinal Healthy Longevity Survey (CLHLS) [15] and China Health and Retirement Longitudinal Study (CHARLS) [16] have explored the health profile of the Han Chinese; the Sukagawa Study focuses on Japanese aged 75 years above [17]. However, it should be highlighted that knowledge on the biological and clinical ageing phenotypes and their determinants among the oldest-old is still limited. Asian populations represent the majority of oldest old in the world, whereas the majority of previous findings are based on Western populations. Asians are different from their Western counterparts in terms of genetics, lifestyle, culture and other determinants [18–20]. The SG90 cohort study attempts to fill some of the important gaps in knowledge by following a cohort of oldest-old Singaporeans.

The primary objective of the SG90 cohort study is to characterize healthy ageing in the oldest-old, identifying a range of modifiable medical, biological, lifestyle, psychological, behavioural, and social factors that may contribute to healthy longevity, thus creating a comprehensive foundation for guiding future intervention studies.

## Methods

### Study design

**Fig. 1:**
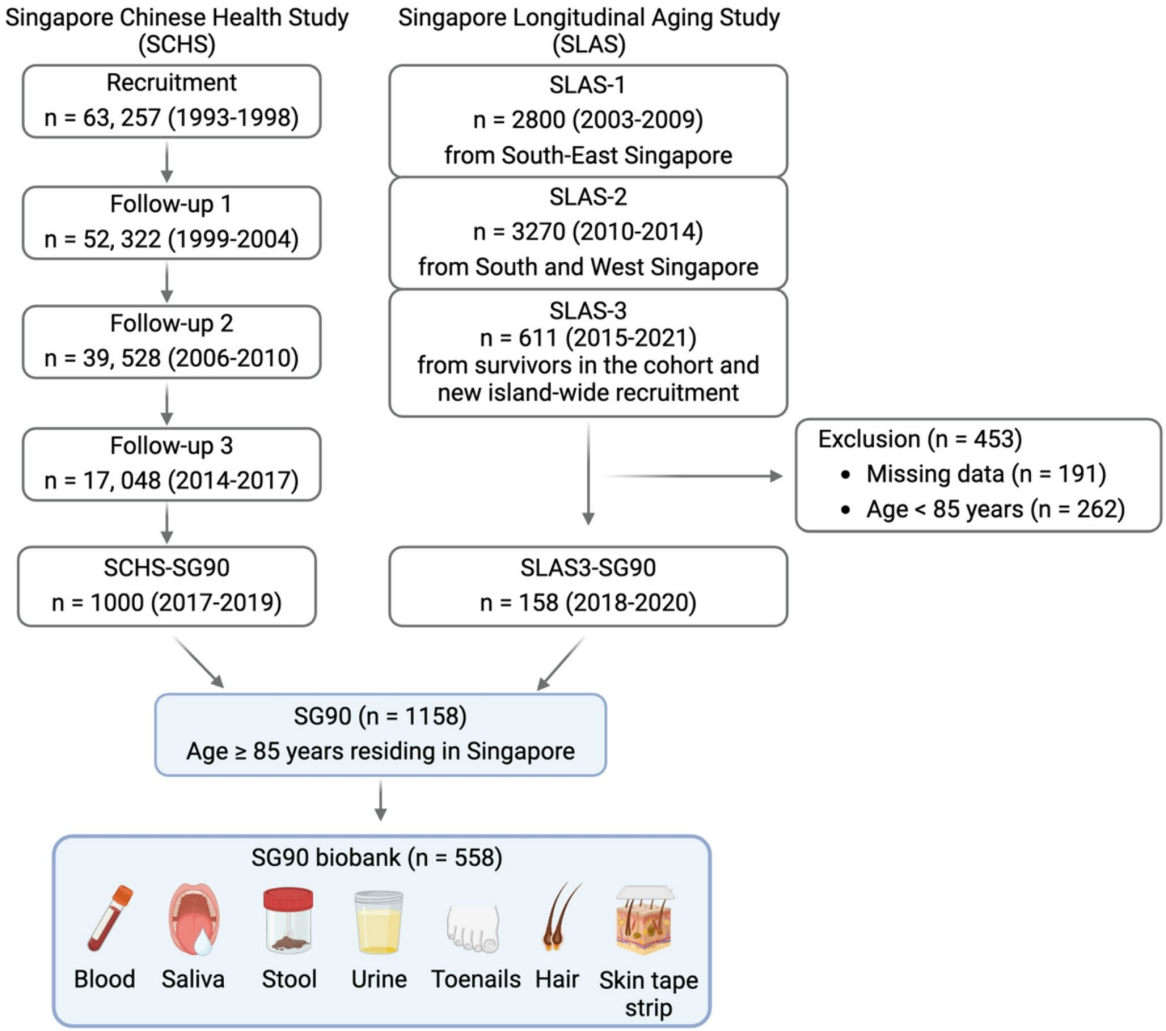
Flowchart of SG90 sub-cohort from Singapore Chinese Health Study (SCHS) and Singapore Longitudinal Aging Study (SLAS) cohorts

The SG90 cohort comprised of subsets of the population-based prospective cohorts Singapore Chinese Health Study (SCHS) participants and Singapore Longitudinal Aging Study (SLAS) participants who were aged 85 years and above. The SCHS is an on-going prospective cohort study designed to evaluate the genetic, dietary, and environmental determinants of chronic diseases in Chinese adults living in Singapore. Detailed descriptions of the study have been reported previously [21]. In brief, 63,257 Chinese participants (27,959 male and 35,298 female) aged 45–74 (mean 53) years old were enrolled between April 1993 and December 1998. After recruitment, the participants were re-contacted for follow-up interviews in 1999–2004 (phone or in-person), 2006–2010 (phone or in-person), and 2014–2017 (in-person). In this SCHS-SG90 sub-cohort, due to limited recourses, the first 1,000 consenting participants who were aged 85 years and above were recruited to participate in SG90 between July 2017 and March 2021 [22]. SLAS is a population-based prospective and longitudinal cohort study on ageing and health of adults aged over 55 years residing in Singapore [23]. Participants were systematically identified through a comprehensive door-to-door census of community-dwelling older adults and then voluntarily invited to participate in the study. Individuals who were unable to participate due to severe physical or mental disabilities were excluded. The recruitment for SLAS-1 occurred between 2003 and 2009 in the South-East region of Singapore (*n* = 2,800), followed by SLAS-2 recruitment from the South and West regions of Singapore in 2010–2014 (*n* = 3,270) and SLAS-3 (aged over 80 years) from survivors and new island-wide recruitment in 2015–2021 (*n* = 611). Besides whole population sampling from these regions in Singapore, SLAS also used a nationally representative sampling list of households with oldest-old individuals provided by the Singapore Sampling Design Service, Department of Statistics. The SLAS-3 participants who were aged over 85 years were invited for further interviews and assessments according to the SG90 study protocol, which formed SLAS3-SG90 sub-cohort (*n* = 158). In total, 1,158 participants were included in SG90 cohort. The flow chart of SG90 recruitment is shown in Fig. 1.

### Data collection

The SG90 data was collected through extensive face-to-face structured interviews, clinical assessments, neuropsychological tests, and biospecimen sampling (Table 1). All procedures were conducted by trained interviewers, research nurses and research assistants at the on-site study senior activity centres or participants’ home.

**Table 1:** Data collection in the SG90 cohort

Components	Measures
Face-to-face interview	
Sociodemographics	Age, gender, ethnicity, religion, education level, marital status, housing type, living situation, employment status, social connectedness
Family information	Information on spouse, offspring, sibling and spouse of sibling
Lifestyle	Substances use (cigarette smoking, alcohol consumption, tea, coffee), Food Frequency Questionnaire (FFQ) [38], physical activities, social activities, leisure-time activities, General Physical Activities Questionnaire (GPAQ) [39]
Sleep quality	Pittsburg Sleep Quality Index (PSQI) [40]
Functional status	Barthel Index Basic Activities of Daily Living (BADL) [41], Lawton and Brody Instrumental Activities of Daily Living (IADL) [42], WHO Disability Schedule (WHODAS) [43]
Quality of life	WHO Quality of Life-AGE (WHOQoL-AGE) [44], Short form (SF)-12 [45], Life Satisfaction Scale (LSS) [46]
Medical conditions	History of falls, chronic diseases, medical procedures, hospitalization, Fracture Risk Assessment Tool (FRAX) [47], Chronic obstructive pulmonary disease (COPD) questionnaire, medications, supplements, family history of chronic diseases, self-rated successful ageing
Healthcare economics	Utilization of health services (general practitioner/family physician, emergency department, inpatients, outpatient, traditional medicine), insurance plans
Clinical assessments	
Physical examination	Blood pressures (supine, sitting, standing)
Anthropometry	Height, weight, waist/hip/mid-calf/thigh/mid-upper-arm circumference, supine total arm length, knee to floor height
Physical performance	Handgrip strength [24], Timed Up and Go test (TUG) [25], 6-meter walk test at fast pace [26], Short Physical Performance Battery (SPPB) [27, 28]
Cognitive function	Mini-Mental State Examination (MMSE) [29, 30], Subjective Memory Cognitive Complaints (SMCC) [31], Informant Rated Memory Decline (IRMD) [32]
Mental health	15-item Geriatric Depression Scale (GDS-15) [33]
Biobanking and biological analysis	
Blood	Clinical laboratory tests, DNA methylation, immunophenotyping, metabolomics, proteomics, lipidomics, RNA sequencing, whole genome sequencing, plasma Toll-Like Receptors (TLR) stimulation, serum analytes
Saliva	Oral microbiome
Stool	Shortgun metagenomics, DNA methylation, RNA sequencing
Urine	Metabolomics, markers of oxidative damage
Toenails	Exposure to toxins
Hair	Exposure to toxins
Skin tape strip	Shortgun metagenomics

#### Face-to-face interview

Participants were visited for a face-to-face interview in their preferred language (e.g. English, Mandarin, Chinese dialects such as Hokkien or Cantonese). A standardized structured questionnaire was used to collect self-reported information on sociodemographics, family information, lifestyle (substances use, diet, physical and leisure-time activities), sleep quality, functional status, quality of life, medical conditions and healthcare economics.

#### Clinical assessments

Blood pressure was measured in supine, sitting and standing positions using an automatic digital blood pressure monitor HEM-705CP (Omron Corporation, Japan). Height (cm) was measured to the nearest 1.0 cm using a rigid, self-retracting metal tape measure, while weight (kg) was recorded to the nearest 0.1 kg with a Soehnle Exacta Comfort digital weighing scale (Model S63315 PSD). Circumference measurements (cm) were obtained using a soft, flexible plastic tape. Waist circumference was measured 2.5 cm (1 inch) above the participant’s navel, while hip, leg, and thigh circumferences were measured at the widest part of the respective region. Mid-upper-arm circumference was measured with the forearm bent at 90 degrees, positioning the tape between the head of the humerus and the olecranon. Supine total arm length (cm) was recorded to the nearest 0.1 cm from the acromion to the olecranon and from the olecranon to the styloid process of the ulna using a soft tape. Knee-to-floor height (cm) was measured with the subject seated, placing a tape measure from the fibular head down to the floor. Handgrip strength was assessed using a Jamar Plus + digital hand dynamometer (Pennsylvania, United States) with both hands tested alternately three times, while the subject was seated with the arm close to the side, elbow at 90 degrees, and wrist in 0-30-degree dorsiflexion and 0-15-degree ulnar deviation [24]. The Timed Up and Go test (TUG) was measured in seconds and conducted on a flat surface over a marked 3-meter distance, with subjects starting seated and instructed to stand, walk to the finish line, return, and sit [25]. The six-meter walking test was performed to measure gait speed (m/s) at fast pace and recorded in seconds [26]. Short Physical Performance Battery (SPPB) consists of a repeated chair stands test, a balance test and the 6-meter walking test at fast pace with a total score from 0 to 12 [27, 28]. Cognitive function was evaluated by Mini-Mental State Examination (MMSE) [29] which has been modified for the use in Singapore [30], Subjective Memory Cognitive Complaints (SMCC) [31], and Informant Rated Memory Decline (IRMD) [32]. The optimal cut-off scores of MMSE for no, primary and secondary and above education levels are 25, 27 and 29, which was validated in a large cohort of Singapore Chinese older adults [30]. The presence of depressive symptoms was assessed using the 15-item Geriatric Depression Scale (GDS-15), a tool validated for use among Singaporean Chinese, Malay, and Indian populations [33].

#### Biobanking and biological analysis

Trained research nurses performed phlebotomy to collect venous blood for both clinical laboratory tests and biological markers of ageing. A total of 48 millilitres of fasting blood was collected into different types of tubes for different research purposes. Blood collected in K2 ethylenediaminetetraacetic acid (EDTA) tubes (BD Vacutainer, NJ, USA), Serum-Separating Tubes (SST) (BD Vacutainer, NJ, USA), Sodium Fluoride tubes (BD Vacutainer, NJ, USA) was sent to the National University Hospital Referral Laboratory (NRL) for clinical laboratory tests including complete blood counts, lipid profiles, albumin, fasting glucose, creatinine, high-sensitivity C-reactive protein (hs-CRP), vitamin B12, folate, plasma total homocysteine, electrolytes (sodium, potassium, calcium), liver enzymes [alanine transaminase (ALT), aspartate transaminase (AST)], and thyroid function markers [free thyroxine, thyroid-stimulating hormone (TSH)]. The rest of blood collected in SST tubes, Tempus™ Blood RNA Tubes (Applied Biosystems, CA, USA) and CPT™ Mononuclear Cell Preparation - Sodium Citrate Tubes (BD Vacutainer, NJ, USA) was sent to Singapore Immunology Network (SIgN) to process into serum, plasma, peripheral blood mononuclear cells (PBMCs), sorted immune cells, and red blood cells and biobank according to established protocols. Additionally, saliva, stool, urine, toenails, hair, and skin tape strip were collected according to standard operation procedures to support a comprehensive view of the biological ageing process (Fig. 1). The other institutes Genome Institute of Singapore (GIS) and the Singapore Institute of Clinical Sciences (SICS) were also involved in ageing biomarkers analyses.

The rich collection of biomaterial supports extensive multi-omic and cellular analyses, which provide deep insights into the biological determinants of ageing. Specifically, DNA methylation analyses assess epigenetic markers associated with biological age, while immunophenotyping enables detailed profiling of immune cell populations and illuminates ageing patterns in immune systems. Multi-omics analyses including metabolomics, proteomics, lipidomics, transcriptomics, and genomics were performed to identify systemic biomarkers across molecular pathways relevant to ageing and resilience. Plasma toll-like receptors (TLR) stimulation and serum analytes assessments are essential to enhance the understanding of immune functions, metabolic health, inflammatory status and nutritional deficiencies. In addition, trace element and heavy metal analysis in toenails and hair samples reveals cumulative exposure to environmental toxins which potentially contribute to chronic diseases and accelerated ageing (Table 1).

#### Follow-up

Mortality data are obtained via record linkage through the National Registry of Disease Office (NRDO) at the Ministry of Health. Morbidity will be captured through Singapore’s National Electronic Health Record (NEHR) system, which provides comprehensive data on incident diagnoses and hospitalizations. To maintain cohort size and statistical power amid high mortality rates in the oldest-old, the SG90 study will implement regular follow-up assessments and consider replenishment recruitment of newly eligible participants.

## Results

The total included 1,158 participants had a median age of 87 years old [interquartile range (IQR): 86–89)] and 64.5% of female. Most participants were of Chinese ethnicity (97.5%) with Taoism or Buddhism belief. No formal education was received in 46.9% of participants with a higher frequency in female. Most participants were widowed (64.1%), with a higher proportion of females than males. Nearly half (47.0%) lived in 4–5 room Housing and Development Board (HDB) flats, and 12.4% lived alone. Most participants were non-smokers (72.1%), never or rarely consumed alcohol (94.9%) and reported often exercising 2–3 times per week (66.5%) (Table 2). Participants tended to be functional independent with a median Barthel Index basic activities of daily living score of 20 (IQR: 19–20) and Lawton and Brody instrumental activities of daily living score of 14 (IQR: 11–16). The quality of life score was relatively higher in males than females with a mean score of 73.3 [standard deviation (SD): 9.5] and 70.8 (SD: 10.5), respectively. 36% of participants rated their self-reported health as good to excellent, while 25.4% of participants reported their health as poor. One quarter of participants experienced falls in the past one year (24.2%) and reported having arthritis (24.6%) and/or diabetes (24.1%). Hypertension was the most common medical condition (76.7%), followed by high cholesterol (66.0%), with a median sitting systolic blood pressure of 139.8 mmHg (SD: 20.2). Men had a median height of 161 cm (IQR: 157–165), mean weight of 58.0 kg (SD: 10.2), and median body mass index (BMI) of 22.4 kg/m² (IQR: 19.7–24.6), indicating that most male participants were within the normal-weight range. Women had a median height of 148 cm (IQR: 144–152), mean weight of 51.8 kg (SD: 10.0), and median BMI of 23.4 kg/m² (IQR: 20.7–26.4), suggesting on average female participants fell within the overweight category according to Asian BMI thresholds. The mean waist circumference was 87.9 cm (SD: 10.4) in men and 87.0 cm (SD: 11.1) in women, indicating that on average men were slightly below, while women exceeded, the Asian abdominal obesity cut-off which is ≥ 90 cm for men, ≥ 80 cm for women. Men showed better physical performance with a higher median handgrip strength (22.2 kg, IQR: 18.4–26.0) and a higher median SPPB score (7, IQR: 5–9) compared to women who had a median handgrip strength of 13.5 kg (IQR: 10.5–16.2) and a median SPPB score of 4 (IQR: 2–6), reflecting expected sex differences in muscle strength and functional capacity among older adults. Participants had a median MMSE score of 23 (IQR: 19–26) and a median GDS-15 score of 3 (IQR: 1–6) indicating mild to moderate cognitive impairment and minimal depressive symptoms (Table 3). The above provides an overview of the descriptive statistics of the SG90 cohort. More findings on specific research questions have been detailed elsewhere [22, 34–37].

**Table 2:** Sociodemographic and lifestyle characteristics of participants in the SG90 cohort

		*n*	Total (*N* = 1158)	Male (*n* = 411)	Female (*n* = 747)
Age, years		1158	87 [86–89]	87 [85–88]	87 [86–89]
Ethnicity		1158			
Chinese			1129 (97.5)	403 (98.1)	726 (97.2)
Malay			12 (1.0)	4 (1.0)	8 (1.1)
Indian			10 (0.9)	2 (0.5)	8 (1.1)
Others			7 (0.6)	2 (0.5)	5 (0.7)
Religion		1149			
Taoist/Buddhist			745 (64.8)	249 (61.5)	496 (66.7)
Christian			262 (22.8)	84 (20.7)	178 (23.9)
Islam			14 (1.2)	4 (1.0)	10 (1.3)
Hindu			4 (0.3)	2 (0.5)	2 (0.3)
Others			124 (10.8)	66 (16.3)	58 (7.8)
Education level		1156			
Nil			542 (46.9)	83 (20.2)	459 (61.4)
Primary			464 (40.1)	227 (55.4)	237 (31.8)
Secondary or equivalent			124 (10.7)	85 (20.7)	39 (5.2)
Pre-university/Polytechnic			22 (1.9)	11 (2.7)	11 (1.5)
University and above			4 (0.3)	4 (1.0)	0 (0)
Marital status		1158			
Single			34 (2.9)	12 (2.9)	22 (2.9)
Married			364 (31.4)	271 (65.9)	93 (12.4)
Divorced/Separated			18 (1.6)	3 (0.7)	15 (2.0)
Widowed			742 (64.1)	125 (30.4)	617 (82.6)
Housing type		1157			
1–2 room HDB			190 (16.4)	60 (14.6)	130 (17.4)
3 room HDB			383 (33.1)	152 (37.0)	231 (31.0)
4–5 room HDB			544 (47.0)	191 (46.5)	353 (47.3)
Executive/Mansionette			15 (1.3)	3 (0.7)	12 (1.6)
Private apartment/Condominium			14 (1.2)	4 (1.0)	10 (1.3)
Landed Property			11 (1.0)	1 (0.2)	10 (1.3)
Living alone		1158	144 (12.4)	42 (10.2)	102 (13.7)
Smoking status		1145			
Non-smoker			826 (72.1)	172 (42.4)	654 (88.5)
Ex-smoker			251 (21.9)	192 (47.3)	59 (8.0)
Current smoker			68 (5.9)	42 (10.3)	26 (3.5)
Alcohol consumption		1146			
Never or rarely			1088 (94.9)	359 (88.4)	729 (97.6)
> 1 drink/month but < 1 drink/week			15 (1.3)	11 (2.7)	4 (0.5)
> 1 drink/week but < 1 drink/day			31 (2.7)	25 (6.2)	6 (0.8)
1–2 drinks a day			8 (0.7)	7 (1.7)	1 (0.1)
3 or more drinks a day			4 (0.3)	4 (1.0)	0 (0.0)
Exercise 2–3 times/week		1155			
Never			361 (31.3)	125 (30.6)	236 (31.6)
Sometimes			26 (2.3)	11 (2.7)	15 (2.0)
Often			768 (66.5)	273 (66.7)	495 (66.4)

Data are presented as n (%), or median [Interquartile range]

*HDB*  The Housing and Development Board

**Table 3:** Characteristics of functional status, quality of life, medical conditions and clinical assessments in the SG90 cohort

		*n*	Total (*N* = 1158)	Male (*n* = 411)	Female (*n* = 747)
Barthel Index BADL		1152	20 [19–20]	20 [19–20]	20 [19–20]
Lawton and Brody IADL		1150	14 [11–16]	15 [13–16]	13 [10–15]
WHOQOL-AGE		1140	71.7 ± 10.2	73.3 ± 9.5	70.8 ± 10.5
Self-reported health		1151			
Excellent			24 (2.1)	10 (2.5)	14 (1.9)
Very Good			89 (7.7)	40 (9.8)	49 (6.6)
Good			301 (26.2)	118 (28.9)	183 (24.6)
Fair			445 (38.7)	157 (38.5)	288 (38.8)
Poor			292 (25.4)	83 (20.3)	209 (28.1)
Falls in the past 1 year, yes		1150	278 (24.2)	95 (23.3)	183 (24.7)
Medical conditions					
Hypertension		1153	884 (76.7)	299 (72.9)	585 (78.7)
High cholesterol		1152	760 (66.0)	266 (65.0)	494 (66.5)
Arthritis		1150	283 (24.6)	67 (16.5)	216 (29.1)
Diabetes		1152	278 (24.1)	98 (24.0)	180 (24.2)
Cardiovascular diseases		1150	230 (20.0)	88 (21.6)	142 (19.1)
Stroke		1149	101 (8.8)	41 (10.1)	60 (8.1)
Osteoporosis		1149	91 (7.9)	13 (3.2)	78 (10.5)
Neurodegenerative diseases		1150	25 (2.2)	8 (2.0)	17 (2.3)
COPD		1150	20 (1.7)	14 (3.4)	6 (0.8)
Sitting blood pressure					
Systolic, mmHg		741	139.8 ± 20.2	138.4 ± 20.9	140.7 ± 19.7
Diastolic, mmHg		741	69.0 [62.0–76.0]	68.0 [62.0–75.0]	70.0 [63.0–76.0]
Height, cm		1085	152 [146–159]	161 [157–165]	148 [144–152]
Weight, kg		1093	54.0 ± 10.5	58.0 ± 10.2	51.8 ± 10.0
Body mass index, kg/m^2^		1082	22.9 [20.4–25.5]	22.4 [19.7–24.6]	23.4 [20.7–26.4]
Waist circumference, cm		743	87.4 ± 10.8	87.9 ± 10.4	87.0 ± 11.1
Handgrip strength, kg		743	16.0 [12.0-20.5]	22.2 [18.4–26.0]	13.5 [10.5–16.2]
SPPB		748	5 [3–8]	7 [5–9]	4 [2–6]
MMSE		1141	23 [19–26]	25 [21–27]	21 [18–24]
Geriatric depression scale-15		1144	3 [1–6]	3 [1–5]	3 [2–7]

Data are presented as n (%), or median [Interquartile range], or mean ± standard deviation

* BADL* Basic activities of daily living, *COPD* Chronic obstructive pulmonary disease, *IADL* Instrumental activities of daily living, *MMSE* Mini mental state examination, *SPPB*  Short physical performance battery, *WHOQOL-AGE* The World Health Organization quality of life in the ageing population

## Discussion

The SG90 cohort focuses on an understudied population, the oldest-old in Southeast Asia, which allows for investigation on the determinants of healthy ageing and development of potential interventions to promote longevity and quality of life. The detailed questionnaire and tests applied cover a broad spectrum of physical, mental and social health-related factors, and provide a comprehensive view of this population’s well-being. Additional extensive biomaterial sampling enables a variety of biological assessments, presenting the participants’ biological profiles but also serving as a valuable biobank for future investigations into ageing processes. Nevertheless, there are some limitations. The cohort may be prone to healthier volunteer bias, as those able to participate and provide biospecimens could be in better health than the general oldest-old population. The recall bias could not be ruled out, particularly from self-reported data on health history and behaviours which might affect the accuracy of some data points. Generalizability may be limited due to the dominance of Chinese participants, with fewer Malay and Indian individuals represented.

## Data Availability

Data sharing policy and procedures were established, and qualified researchers with proven track records and specific research questions are welcome to conduct secondary data analysis from the cohort with research collaboration agreements. For further information, please contact the corresponding author.
